# Green Synthesized Zinc Oxide (ZnO) Nanoparticles Induce Oxidative Stress and DNA Damage in *Lathyrus sativus* L. Root Bioassay System

**DOI:** 10.3390/antiox6020035

**Published:** 2017-05-18

**Authors:** Kamal K. Panda, Dambaru Golari, A. Venugopal, V. Mohan M. Achary, Ganngam Phaomei, Narasimham L. Parinandi, Hrushi K. Sahu, Brahma B. Panda

**Affiliations:** 1 Molecular Biology and Genomics Laboratory, Department of Botany, Berhampur University, Berhampur 760007, Odisha, India; kamalpanda51@gmail.com (K.K.P.); dambarugolari@gmail.com (D.G.); venu86job@gmail.com (A.V.); 2 Plant Molecular Biology Group, International Centre for Genetic Engineering and Biotechnology, New Delhi 110067, India; achary.mohan1@gmail.com; 3 Material Chemistry Laboratory, Department of Chemistry, Berhampur University, Berhampur 760007, Odisha, India; g.phaomei@gmail.com; 4 Division of Pulmonary, Allergy, Critical Care, and Sleep Medicine, Department of Medicine, Davis Heart and Lung Research Institute, Ohio State University College of Medicine, Columbus, OH 43210, USA; narasimham.parinandi@osumc.edu; 5 Condensed Matter Physics Division, Indira Gandhi Centre for Atomic Research, Kalpakum, Tamil Nadu 603102, India; sahuhk@gmail.com

**Keywords:** zinc oxide nanoparticles, green synthesis, *Calotropis gigantea*, plant bioassay, *Lathyrus sativus* root, oxidative stress, comet assay, reactive oxygen species

## Abstract

Zinc oxide nanoparticles (ZnONP-GS) were synthesised from the precursor zinc acetate (Zn(CH_3_COO)_2_) through the green route using the milky latex from milk weed (*Calotropis gigantea* L. R. Br) by alkaline precipitation. Formation of the ZnONP-GS was monitored by UV-visible spectroscopy followed by characterization and confirmation by energy-dispersive X-ray spectroscopy (EDX), transmission electron microscopy (TEM), and X-ray diffraction (XRD). Both the ZnONP-GS and the commercially available ZnONP-S (Sigma-Aldrich) and cationic Zn^2+^ from Zn(CH_3_COO)_2_ were tested in a dose range of 0–100 mg·L^−1^ for their potency (i) to induce oxidative stress as measured by the generation reactive oxygen species (ROS: O_2_^•−^, H_2_O_2_ and ^•^OH), cell death, and lipid peroxidation; (ii) to modulate the activities of antioxidant enzymes: catalase (CAT), superoxide dismutase (SOD), guaiacol peroxidase (GPX), and ascorbate peroxidase (APX); and (iii) to cause DNA damage as determined by Comet assay in *Lathyrus sativus* L. root bioassay system. Antioxidants such as Tiron and dimethylthiourea significantly attenuated the ZnONP-induced oxidative and DNA damage, suggesting the involvement of ROS therein. Our study demonstrated that both ZnONP-GS and ZnONP-S induced oxidative stress and DNA damage to a similar extent but were significantly less potent than Zn^2+^ alone.

## 1. Introduction

At present, metal oxide nanoparticles (NPs), owing to their optical, electrical, and magnetic properties, are sought after materials for applications in the fields of energy storage, sensors, data storage, optics, transmission, cosmetics, biotechnology, and medicine. To meet the growing demand for NPs, they are manufactured in large scale for industrial and household uses. The increasing use of NPs leads to their release into the environment and serious risk of ecotoxicity [1,2,3]. Hence, metal oxide NPs emerge as a novel class of environmental xenobiotics, the toxicity/hazard of which needs to be assessed on a priority basis. Furthermore, plants, as lifeforms of the first trophic level, are at the forefront of encountering environmental NPs. Therefore, there is a pressing need to obtain vital knowledge on the adverse effects of NPs on plants towards obtaining the crucial environmental impact assessment of NPs. 

Among metal oxide NPs, zinc oxide nanoparticles (ZnONPs) with unique optical and electrical properties are suitable for many applications such as coatings for the solar cells, electronics, and chemical sensors. The ZnONP blocks the UV-radiation and therefore is used in transparent UV-protection films and as a UV-filter in sunscreens [4]. Due to its antimicrobial properties, ZnONP is included in the linings for canning meat, fish, corn, and peas [5]. Recent studies demonstrate that ZnONPs hold considerable promise for biomedical applications and therapeutic intervention [6]. The ZnONP-amended agricultural soils depending on the soil-properties cause phytotoxicity in wheat seedlings [7]. Therefore, the ecotoxicity of the ZnONPs in diverse organisms is of serious concern [8]. 

Many alternative physico-chemical [9,10,11,12,13] and biological or plant-mediated [14,15] synthesis routes have been used to prepare ZnONPs. The latter methods using microbes and plant extracts are preferred for the synthesis of NPs, which are considered eco-friendly. The relatively simple bottom-up approach using the plant extracts, also known as green synthesis (GS), minimizes or eliminates hazardous chemicals normally used in physico-chemical synthesis of NPs. Furthermore, GS is preferred for the synthesis of metallic NPs due to reasonable (low) cost, less or no toxicity, and other environmental advantages [16]. GS of the ZnONP utilizes the extracts of different plants, including *Acalypha indica*, *Aloe vera*, *Calotropis gigantea*, *C. procera*, *Catharanthus roseus*, *Coriandrum sativum*, and *Ixora coccinia* [17,18,19,20,21], as well as extracts of the algal seaweeds [22]. Among currently available methods of GS, the present study utilized the alkaline precipitation method to synthesize the ZnONP by reduction of the precursor, zinc acetate (Zn(CH_3_COO)_2_), using sodium hydroxide (NaOH) along with the milky latex of *C. gigantea*. Furthermore, the potential of ZnONP synthesized by the green route (henceforth referred as ZnONP-GS) to induce oxidative stress and DNA damage was determined in comparison with that of the commercially available ZnONP-S (Sigma-Aldrich, St. Louis, MO, USA) and Zn^2+^ ion from Zn(CH_3_COO)_2_. In order to investigate the role of reactive oxygen species (ROS) in the phytotoxicity of the ZnONPs, well-established antioxidants such as Tiron (1,2-dihydroxybenzene-3,5-disulfonate) and dimethylthiourea (DMTU), which scavenge O_2_^•−^ and H_2_O_2_ respectively, were used in this study. Oxidative and DNA damage assays were carried out by employing the *Lathyrus* *sativus* L. root bioassay system [23]. 

## 2. Materials and Methods

### 2.1. Synthesis of ZnONP

Fresh milky latex was collected by making an incision on the intact branches of a mature milk weed, *Calotropis gigantea* (L.) Dryand (Family: Apocyanacae, Subfamily: Asclepiadoideae). The freshly collected milky latex in different volumes 0.25, 0.5, or 1.0 mL was added to 100 mL aqueous solution of 0.01 M zinc acetate, Zn(CH_3_COO)_2_ 2H_2_O (Merck, Mumbai, India), with constant stirring at room temperature (25 ± 1 °C) for thorough mixing (1:100, by vol.). The initial pH recorded in the range of 7.6–7.8 was raised to pH 12 by adding 2 M NaOH (~2.4 mL), which resulted in the appearance of white precipitation of ZnONP-GS within 10 min. The ZnONP-GS was continuously mixed in the synthesis container for 2 h and then allowed to settle overnight at room temperature. The photoluminescence spectrum of the clear supernatant, owing to the surface plasmon resonance (SPR) of the clear supernatant obtained by centrifugation, was recorded in the wavelength range of 200 to 600 nm at a resolution setting of 0.1 nm on a UV-visible spectrophotometer (UV-3000+, LabIndia Instruments, Mumbai, India). The pellet of ZnONP-GS was collected by centrifugation at 12,000 rpm for 15 min followed by repeated washings with double distilled water and ethanol to remove the residual Zn^2+^ ion and other impurities. The absence of any free Zn^2+^ ion in the final wash was ascertained indirectly by measuring the conductivity with the aid of a conductivity meter (YSI Model 85, Yellow Springs, OH, USA). Finally, the ZnONP-GS were air dried for 48 h to a dry power. The absorbance of the supernatant from the ZnONP-GS suspension prepared using distilled water was also recorded for determination of SPR of the pure ZnONP-GS. 

### 2.2. Physical Characterisation of ZnONP 

The green synthesized dry powder of the ZnONP-GS and the commercially available zinc oxide nanoparticles (ZnONP-S) (S: for Sigma-Aldrich, St. Louis, MO, USA) were subjected to X-ray Diffraction (XRD) for identification of the hexagonal wurtzite crystalline solid particles. The samples were smeared on a high index (911) Si plate, which was spun continuously to reduce the effect of any preferred orientation of the particles. The measurements were carried out on a Stoe diffractometer using Cu Kα radiation operating at 45 kV and 40 mA. A coarse ZnO powder with a grain size of ≥25 μm diameter (ZnO Bulk) was used to find out the instrument broadening, and this was used for correcting the actual sample data.

For Transmission Electron Microscopy (TEM), samples of dry powder (~1 mg) of the ZnONPs were mixed with ethanol in a 2 mL Eppendorf tube. The tubes were then placed in an ultrasonic bath for 10 min to disperse the NPs into a uniform suspension. A drop of the suspension was applied onto a 300 mesh copper grid coated with carbon film and dried for 10–30 min in ambient air, and the copper grid was subjected to a TEM examination (JEOL, JEM-2100F, USA operated at 200 kV). The sample was further analysed using Energy-Dispersive X-ray Spectroscopy (EDX, Bruker, Billerica, MA, USA) equipped with a liquid nitrogen free XFlash^®^ Detector at an energy resolution of 127 eV, accelerating voltage of 15 kV, beam current 20 nA, input count rate of 150,000 cps, acquisition time of 5 min, and mapping resolution of 600 × 450 pixels. Also, the dry powder samples of milky latex and that of the latex-synthesizing mixture containing the ZnONP-GS were analyzed by Fourier Transform Infrared Spectroscopy (FTIR) using an IRPrestige-21 FTIR Spectrophotometer (Shimadzu, Nakagyo-ku, Kyoto, Japan) for the identification of functional groups, as described earlier [23].

### 2.3. Plant Root Bioassay System

The seeds of grass pea (*Lathyrus sativus* L., 2*n* = 14) obtained from a local farm were used as the bioassay system. The seeds were surface sterilized in aqueous solutions of a 1% fungicide, Bavistin (BASF, Mumbai, India), thoroughly washed in sterile water, and pre-soaked for 48 h at 4 °C in distilled water to facilitate imbibition. Following complete imbibition, the seeds were wrapped in sterile moist cotton cloth and placed at 24 ± 1 °C in the dark for germination. After 48 h, the germinating seeds with ~15 mm long roots were treated with the experimental solutions at different concentrations [23]. 

### 2.4. Experimental Solutions and Treatment Protocol

Experimental solutions of the ZnONPs were prepared by suspending known amounts (wt.) of the ZnONP-S and the ZnoNP-GS in distilled water under constant stirring for 1 h at different concentrations (0, 10, 20, 40, 80, 100 mg·L^−1^). Solutions of Zn(CH_3_COO)_2_ (Zn^2+^) were likewise prepared in the above range of concentrations to serve as the positive control. The pH of the experimental solutions was adjusted to 7.0 prior to treatment. The germinating seeds were treated by immersing the sprouting roots in 150 mL of the experimental solutions at different concentrations in sterile plastic receptacles (250 mL capacity) under continuous shaking on a rotary shaker for 15 h. In other experiments, to demonstrate the involvement of ROS, germinating seeds were pre-treated (primed) with the O_2_^•−^ scavenger, Tiron (Sigma-Aldrich, USA) and H_2_O scavenger, DMTU (Sigma-Aldrich, USA) at concentrations of 10 or 20 μM for 5 h before the treatment with the ZnONP at 80 mg·L^−1^ for the next 10 h. For all the treatments, double distilled water was used as the negative control (0). The experiments were conducted at room temperature (25 ± 1 °C) in the dark. At the end of the treatments, the germinating seeds with roots were thoroughly washed in running tap water and then processed for cytochemical visualization and spectrophotometric determination of the ROS (O_2_^•−^, H_2_O_2_ and ^•^OH), cell death, lipid peroxidation, antioxidant enzymes activity, and DNA damage by Comet assay in the root tissue. 

### 2.5. Cytochemical Visualization and Spectrophotometric Determination of ROS, Cell Death, and Lipid Peroxidation

The cellular generation of the ROS (O_2_^•−^, H_2_O_2_, ^•^OH) and cell death were visualized by the cytochemical staining techniques and determined spectrophotometrically, as described earlier in detail [23]. The cytochemical detection of lipid peroxidation was performed by staining the root tissue with Schiff’s reagent [24]. Furthermore, lipid peroxidation was determined spectrophotometrically as malondialdehyde (MDA) formed from the ROS-catalyzed membrane polyunsaturated fatty acid peroxidation by the thiobarbituric acid (TBA) reaction [25]. The amount of MDA-TBA complex (red pigment) was calculated from the extinction coefficient (ε = 155 mM^−1^·cm^−1^) and expressed as n moles MDA·g^−1^ fresh weight (FW).

### 2.6. Extraction of Soluble Protein and Enzyme Assays

The root samples (1 g) from each treatment were homogenized in 2 mL of 50 mM Tris-HCl buffer (pH 7.2) containing 0.1 mM ethylenediaminetetraacetic acid (EDTA) and 1% (*w*/*v*) polyvinyl polypyrrolidone at 4 °C. For the ascorbate peroxidase assay, the homogenization solution additionally contained 5 mM ascorbate. The homogenates were centrifuged at 10,000× *g* for 15 min at 4 °C, and the resultant crude supernatant was collected and stored at −20 °C for the determination of protein and enzyme activities. The soluble protein content in the supernatant was determined by the Bradford method with bovine serum albumin as the standard [26]. 

The activities of antioxidant enzymes including catalase (CAT, EC. 1.11.1.6) [27], superoxide dismutase (SOD, EC. 1.15.1.1) [28], guaiacol peroxidase (GPX, EC. 1.11.1.7) [29], and ascorbate peroxidase (APX, EC. 1.11.1.11) [30], were determined spectrophotometrically as described earlier [31]. 

### 2.7. Comet Assay

For the alkaline comet assay, following the 15 h treatment, the germinating seeds with intact roots were removed from the treatment solutions followed by a thorough wash in distilled water. Then the ~15 mm long roots were excised from the seeds and transferred to the glass slide on a 60-mm Petri dish on ice. Then the roots were immersed in 200 μL of chilled Tris-HCl buffer (0.4 M, pH 7.4) and sliced, and the nuclei were collected and processed for the Comet assay using an Olympus BX51 microscope with a fluorescence attachment (using the excitation filter of 515–560 nm and barrier filter of 590 nm) equipped with a Cohu camera and Kinetic Komet^TM^ Imaging Software 5.5 (Andor^TM^ Technology, Belfast, UK, www.andor.com) according to the procedure described earlier [23]. Two slides prepared with the nuclear suspension obtained by slicing at least 15 primary roots (excised from 15 germinated seeds) were examined for each treatment. At least 50 randomly chosen comets were analyzed from each slide. The Comet assay was repeated three times so that a total of 300 comets from six slides prepared from 45 roots (45 germinated roots) were examined for each treatment. The comet images were visualized and captured at 100× magnification. Out of several parameters available in the software, the comets were analyzed based on the percentage (%) DNA in tail as the measure of primary DNA damage [32]. The entire process of Comet assay was carried out in subdued or yellow light. 

### 2.8. Statistical Analysis

All the experiments with the exception of the Comet assay, conducted in triplicate, were repeated once (*n* = 6). The Comet assay was repeated three times (*n* = 6). The pooled data were statistically analyzed for the analysis of variance (ANOVA), followed by Tukey’s honestly significant difference (HSD) test [33] by employing Microsoft Excel 2003 or MATLAB software under the Windows^®^ 7 platform. 

## 3. Results

### 3.1. Synthesis and Characterization of ZnoNP-GS

**Figure 1:**
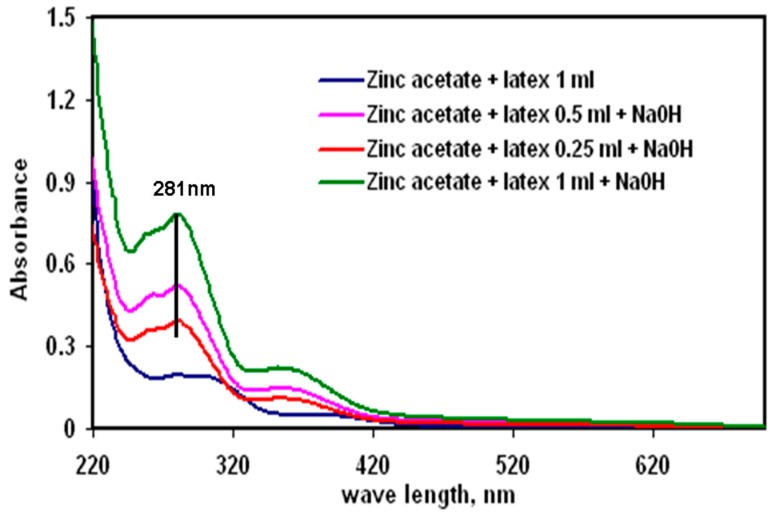
UV-Visible spectrum of a zinc oxide nanoparticle (ZnONP) synthesized by alkaline precipitation method using milky latex from *C. gigantea* at three different concentrations mixed with 100 mL of 0.1 M Zn(CH_3_COO)_2_ solution to which 2 M NaOH solution was added drop wise under stirring to raise the pH to 12.

**Figure 2:**
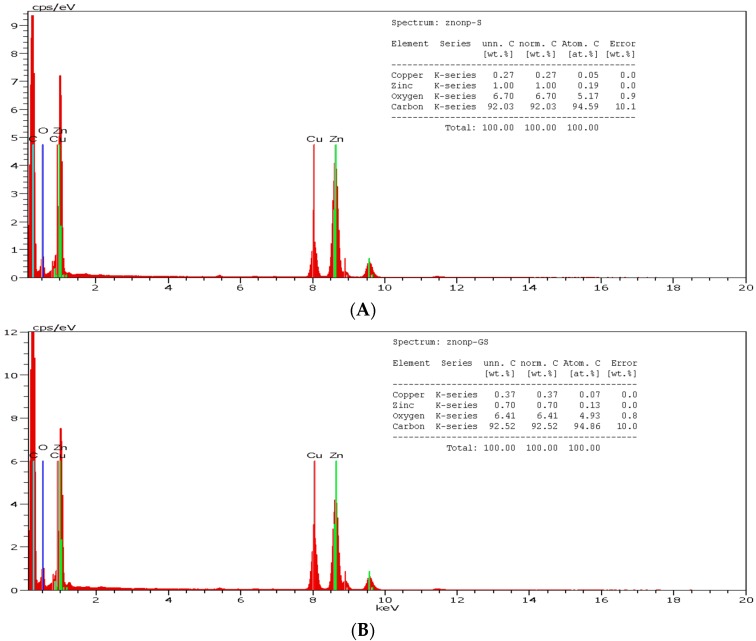
Energy-Dispersive X-ray Spectroscopy (EDX) spectrum of (**A**) ZnONP-S (procured from Sigma-Aldrich, USA) and (**B**) ZnONP-GS (green-synthesized in the current study) showing strong Zn-signals against background signals of C, O, and Cu.

**Figure 3:**
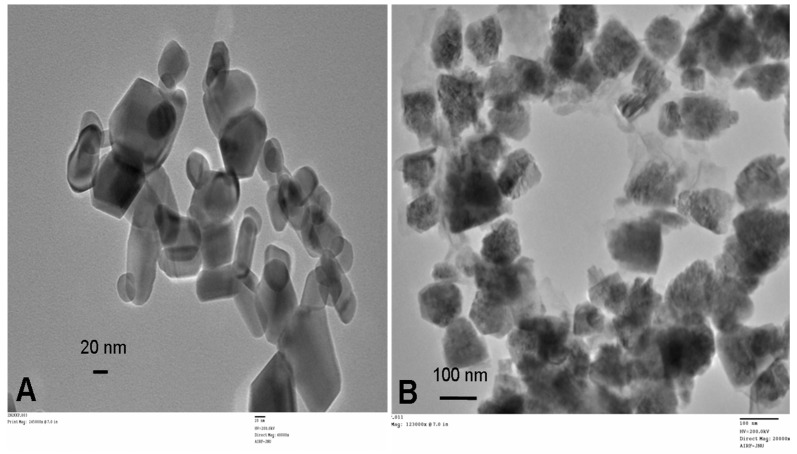
Transmission Electron Microscopy (TEM) images of (**A**) ZnONP-S and (**B**) ZnONP-GS.

**Figure 4:**
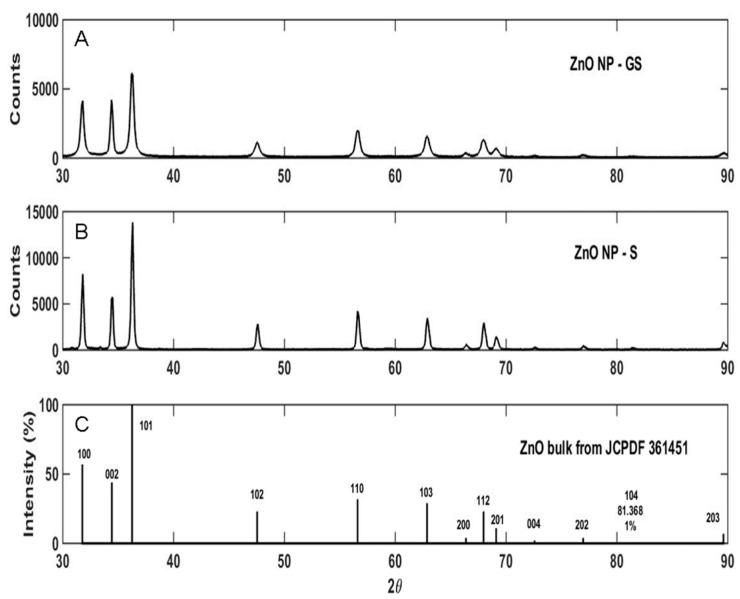
X-ray diffraction (XRD) pattern showing wurtzite crystalline structure of (**A**) ZnONP-GS and (**B**) ZnONP-S compared to (**C**) ZnO Bulk (JCPDS-36-1451).

**Figure 5:**
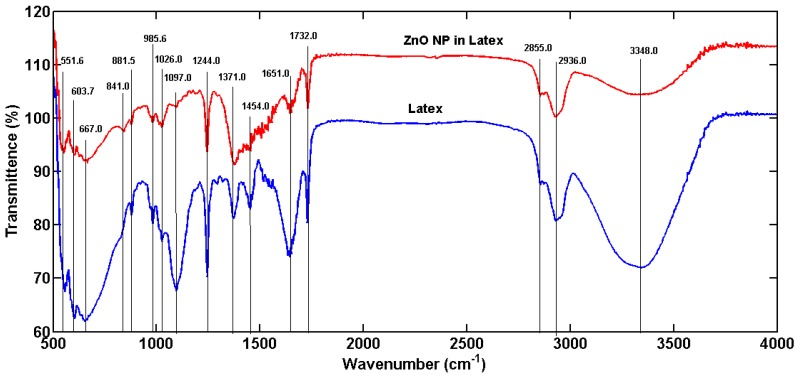
Fourier Transform Infrared (FTIR) spectra recoded from KBr pellets containing dry powder samples of ZnONP-GS in latex (red) and milky latex (blue) from *C. gigantea*.

The raising of the pH of the latex-Zn(CH_3_COO)_2_ mixture to 12 by the addition of 2 M NaOH facilitated the synthesis of ZnONP-GS that was noticeable by the white precipitation. The absorbance was monitored at the wavelength range of 200–600 nm, which showed maximum absorbance (λ_max_) at 281 nm (Figure 1). The λ_max_ observed for the pure ZnONP-GS was 306 nm. The EDX profile showed a strong signal for Zn along with signals of Cu, C, and O (Figure 2). TEM images of the ZnONP-GS and ZnONP-S indicated their spherical to hexagonal shapes (Figure 3). The XRD profile of the ZnONP-GS also agreed well with that of the ZnONP-S used for comparison as well as with that of the standard ZnO bulk (Joint Committee on Power Diffraction Standards, JCPDF-36-1451) (Figure 4), which showed thirteen characteristic diffraction peaks of 100, 002, 101, 102, 110, 103, 200, 112, 201, 004, 202, 104, and 203, observed at 2θ angles; 31.77°, 34.42°, 36.25°, 47.54°, 56.6°, 62.86°, 66.38°, 67.96°, 69.09°, 72.56°, 76.95°, 81.37°, and 89.6°, respectively. The wurtzite crystalline structure of ZnONPs was thus established in conventionality with the JCPDS-36-1451 for ZnO bulk. The sizes of the ZnONP-GS and ZnONP-S were calculated by applying the Scherrer formula, taking into account the first four intense peaks, namely, the 100, 002, 101, and 102 diffraction peaks fitted to the Lorentzian peak shapes, and the diameters were estimated to be 25.1 and 48.6 nm, respectively. The Fourier Transform Infrared (FITR) Spectroscopy profile of *C. gigantea* latex vis-à-vis the ZnONP-GS in latex (Figure 5) revealed the spectral differences marked by the absence of several peaks in the wavenumber range from 1600 cm^−1^ to 1400 cm^−1^ in ZnONP-GS (GS: green synthesis) found prominently in the spectrum of latex.

### 3.2. ROS Generation, Cell Death, and Lipid Peroxidation

At the outset, in a pilot experiment, it was necessary to rule out that the latex at 1% concentration induced any significant effect on the biochemical or biological endpoints measured in the present study. After establishing the fact that the latex at the aforesaid concentration, comparable to distilled water, was non-toxic, in all the subsequent experiments only distilled water was used to serve as the negative control. 

**Figure 6:**
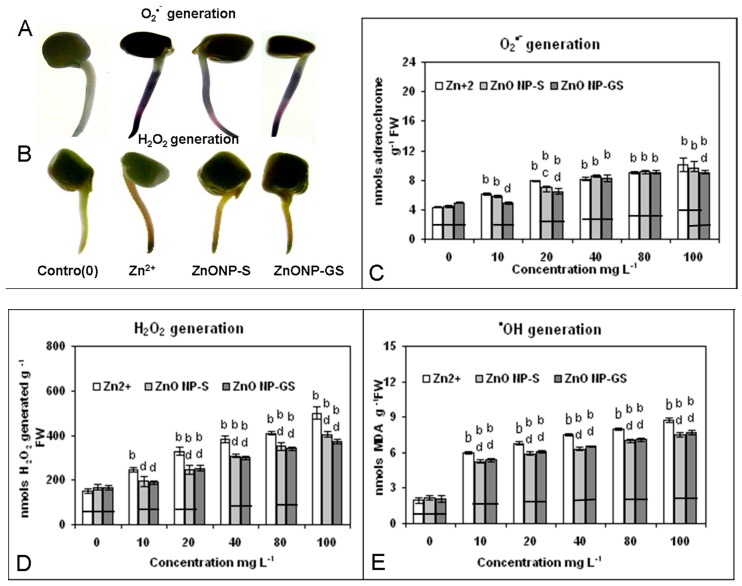
Cytochemical visualization of (**A**) O_2_^•−^, (**B**) H_2_O_2_ in roots of germinating seeds treated with Zn^2+^ or AnONPs at a single concentration (0 or 80 mg·L^−1^); spectrophotometric determination of (**C**) O_2_^•−^, (**D**) H_2_O_2_, and (**E**) ^•^OH in roots of germinating seeds of *C. gigantea* treated with Zn^2+^ or ZnONPs at different concentrations (0–100 mg·L^−1^). Increase significant at *p* ≤ 0.01 (b) compared to the control (0), and decrease significant at *p* ≤ 0.05 (c) or 0.01 (d) compared to Zn^2+^. The horizontal bar denotes no significant difference between the treatments at *p* ≤ 0.05, (*n* = 6).

**Figure 7:**
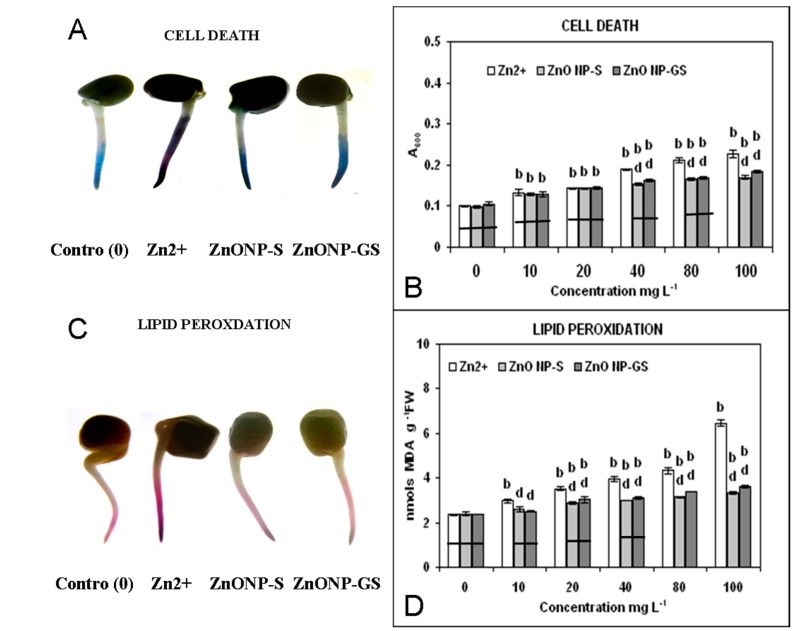
Cytochemical visualization of (**A**) cell death and (**C**) lipid peroxidation in roots of germinating seeds treated with Zn^2+^ and ZnONPs at a single concentration (0 or 80 mg·L^−1^); Spectrophotmetric determination of (**B**) cell death and (**D**) in roots of germinating seeds of *C. gigantea* treated with Zn^2+^ or ZnONPs at different concentrations (0–100 mg·L^−1^). Increase significant at *p* ≤ 0.01 (b) compared to the control (0), and decrease significant at *p* ≤ 0.01 (d) compared to Zn^2+^. The horizontal bar denotes no significant difference between the treatments at *p* ≤ 0.05, (*n* = 6).

The cytochemical visualization and spectrophotometric analysis revealed that Zn^2+^, ZnoNP-S, and ZnONP-GS enhanced the generation of ROS: O_2_^•−^, H_2_O_2_, and ^•^OH (Figure 6) were associated with the concomitant induction of cell death and lipid peroxidation in a dose-dependent fashion (Figure 7). Among the three agonists tested, Zn^2+^ was found to be the most effective to induce significantly the formation of ROS (*p* ≤ 0.01), as revealed from the dose-dependent increase in the generation of O_2_^•−^ (R^2^ = 0.81), H_2_O_2_ (R^2^ = 0.97), or ^•^OH (R^2^ = 0.96), followed by the ZnONP-S (R^2^ = 0.83, 0.97, 0.96) and ZnONP-GS (R^2^ = 0.81, 0.99, 0.93). Although the induction of cell death was observed at concentrations 10 and 20 mg·L^−1^, the difference was evident at concentrations ≥40 mg·L^−1^. For lipid peroxidation, this difference was strikingly evident from the dose of 10 mg·L^−1^ onwards, underscoring Zn^2+^ as more effective than both ZnONP-S and ZnOMP-GS in inducing ROS generation, cell death, and lipid peroxidation.

### 3.3. Activity of Antioxidant Enzymes

**Figure 8:**
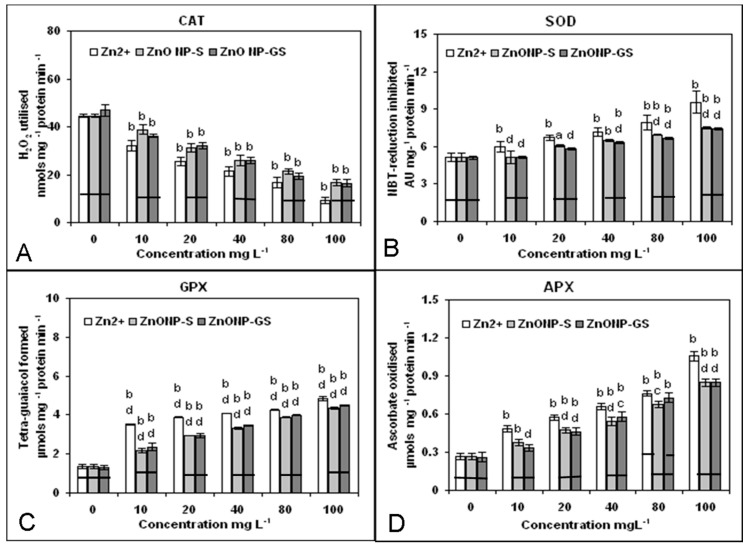
Activity of antioxidant enzymes (**A**) catalase (CAT), (**B**) superoxide dismutase (SOD), (**C**) guaiacol peroxidase (GPX), and (**D**) ascorpbate peroxidase (APX) in the root tissue of germinating seeds of *C. gigantea* treated with Zn^2+^ or ZnONPs at different concentrations (0–100 mg·L^−1^). Increase significant at *p* ≤ 0.05 (a) or 0.01 (b) compared to the control (0), and decrease significant at *p* ≤ 0.05 (c) or 0.01 (d) compared to Zn^2+^. The horizontal bar denotes no significant difference between the treatments at *p* ≤ 0.05, (*n* = 6).

The antioxidative enzymes responded differently to Zn^2+^, ZnONP-S, and ZnONP-GS (Figure 8). Catalase activity was significantly (*p* ≤ 0.01) inhibited by Zn^2+^ (R^2^ = 0.96), ZnONP-S (R^2^ = 0.99), and ZnONP-GS (R^2^ = 0.97) showing a dose-response trend. In contrast, Zn^2+^, ZnONP-S, and ZnONP-GS significantly induced (*p* ≤ 0.01) the activities of SOD (R^2^ = 0.96, 97, 96), GPX (R^2^ = 0.77, 97, 98), and APX (R^2^ = 0.95, 98, 99) in a dose-dependent manner. Once again, Zn^2+^ appeared to be more effective than both the ZnONP-S and ZnOMP-GS in altering the activities of antioxidant enzymes. 

### 3.4. Assessment of DNA Damage

**Figure 9:**
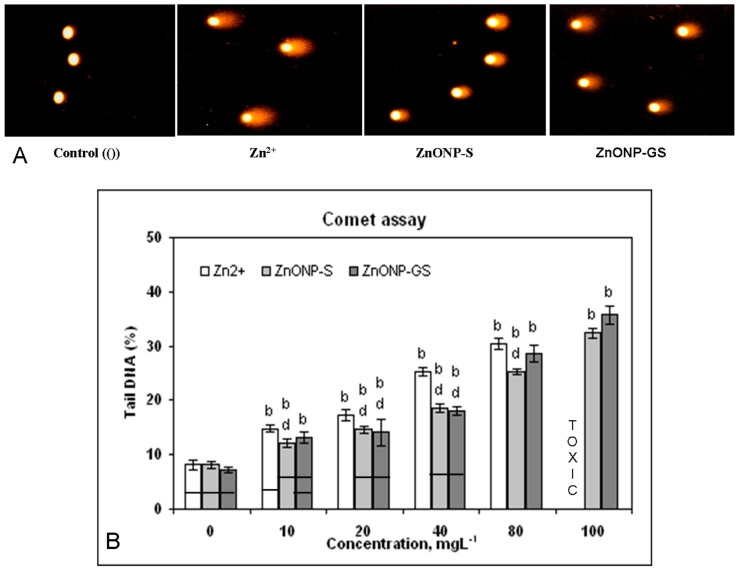
(**A**) DNA damage visualized in form of Comets in root cells from germinating seeds of *C. gigantea* treated with Zn^2+^ or ZnONPs at a single concentration, 0 or 40 mg·L^−1^; (**B**) Dose-dependent induction of DNA damage by Zn^2+^ or ZnONPs at a range of concentrations (0–100 mg·L^−1^). Increase significant at *p* ≤ 0.01 (b) compared to the control (0), and decrease significant at *p* ≤ 0.01 (d) compared to Zn^2+^. The horizontal bar denotes no significant difference between the treatments at *p* ≤ 0.05, (*n* = 6).

The Comet assay that determines the DNA damage based on Tail DNA (%) revealed that Zn^2+^ (R^2^ = 0.98), ZnONP-S (R^2^ = 0.96), and ZnONP-GS (R^2^ = 0.93) significantly induced DNA damage (*p* ≤ 0.01) in a dose-dependent manner (Figure 9). However, Zn^2+^ at the dose of 100 mg·L^−1^ was toxic, as revealed by the comets showing nuclear disintegration or necrosis. 

### 3.5. Effect of Tiron and DMTU on ZnONP-Induced ROS Generation, Cell Death, and DNA Damage

**Figure 10:**
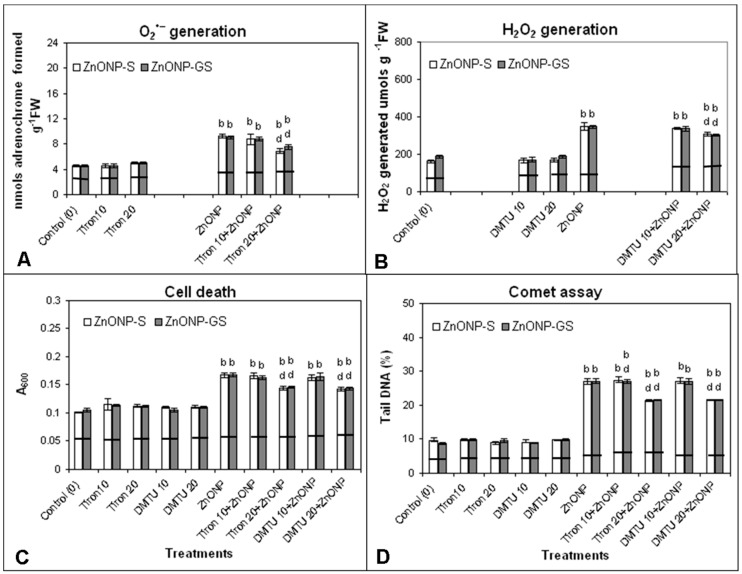
Effect of Tiron- or DMTU-priming at concentrations of 10 and 20 μM on the induction of (**A**) O_2_^•−^ generation, (**B**) H_2_O_2_ generation, (**C**) cell death, and (**D**) DNA damage in roots of germinating seeds of *C. gigantea* treated with ZnONP-GS at 80 mg·L^−1^. Increase significant at *p* ≤ 0.01 (b) compared to the control (0), and decrease significant at *p* ≤ 0.01 (d) compared to ZnONP-GS. The horizontal bar denotes no significant difference between the treatments at *p* ≤ 0.05, (*n* = 6).

The ROS scavengers, Tiron and DMTU (10 and 20 μM), did not appear to alter the generation of O_2_^•−^ and H_2_O_2_ (Figure 10). However, the priming of the roots with Tiron and DMTU (10 μM) had little or no effect on the ROS generation, but the priming of the roots with both scavengers at the dose of 20 μM significantly attenuated the generation of O_2_^•−^ and H_2_O_2_, cell death, and DNA damage that was induced by both the ZnONPs at the dose of 80 mg·L^−1^ (*p* ≤ 0.01).

## 4. Discussion

### 4.1. Green Synthesis and Characterization of ZnONP-GS

The UV-visible spectrophotometry employed in the present study was a preparatory analysis to monitor the synthesis of ZnONP in the crude reaction mixture. The signal (λ_max_) observed at 281 nm, therefore, might have been contributed by other soluble impurities or other side products formed along with the synthesis of ZnONP (Figure 1). The λ_max_ observed separately for the pure ZnONP-GS synthesized in the present study was at 306 nm, which suggested a blue shift in absorption as compared with the bulk of the ZnO (360 nm) and was attributed to the quantum confinement property of NPs, indicating a decrease in the particle size [11,13]. It may be clarified that UV absorption by NPs does not refer to atomic or molecular vibration, but this type of absorption is through a cooperative phenomenon called surface plasmon resonance or SPR. This SPR absorption, therefore, depends on the surface curvature and particle size [11]. The TEM analysis in the present study indicated that the ZnONP-GS sample had a range of shapes from spherical to hexagonal (Figure 3). 

The first step in this process involves breaking down Zn(CH_3_COO)_2_ to form Zn(OH)_2_ by the action of NaOH. At a pH of 12, Zn(OH)_2_ is the prominent species in solution, which then generates ZnO nuclei followed by ZnO crystal growth [11,12]. The intensity of peaks in XRD profiles indicated the Wurtzite crystalline nature of the ZnONP-GS, as with the ZnONP-S; the XRD peak positions corresponded to those reported in the JCPDS 361451 page (Figure 4). The 100% intense peaks were subjected to Scherrer analysis, and the particle sizes were found to be 25.1 nm and 48.6 nm in the ZnONP-GS and ZnONP-S samples, respectively. The FTIR spectrum of the latex of *C. gigantea* without or with the ZnONP-GS (Figure 5) revealed absorption peaks at 3348 cm^−1^ due to OH stretching vibration and at 2855, 2936 cm^−1^ due to –CH_2_– stretching vibration. The characteristic peaks at 1732 cm^−1^ (C=O stretching), 1651 cm^−1^ (N–H bending), 1454 cm^−1^ (C–H bending), 1371 cm^−1^ (C–H rocking), and 1244 cm^−1^ (ester carbonyl) and the peaks at 1097 cm^−1^, 1026 cm^−1^, and 985.6 cm^−1^ (C–N of primary amines) clearly indicate the presence of lupeol, calotropin, calotoxin, and uscharidin, the latex proteins [34,35,36]. The above spectral features associated with the ZnONP-GS in latex seem to be as a result of the binding of latex molecules or proteins to the surface of ZnONPs, serving as capping and stabilizing agents [37]. The addition of latex at such a low concentration as 1% did not introduce any impurity to the ZnONP, as revealed by the XRD analysis reported here. The faint shadow-like contrast around the NPs in TEM rather confirms the capping action of the latex. Ha et al. [11] have reported the synthesis of the ZnONPs from precursors, Zn(CH_3_COO)_2_ and NaOH, by using a conventional heating process or with the aid of microwave irradiation. Furthermore, they have been able to alter the shape by capping the ZnONPs by using surfactants like sodiumdodecyl sulphate (SDS), hexadecylcetyltrimethyl ammonium bromide (CTAB), or polyvinyl pyrrolidone (PVP). The results depicted in Figure 1 led to the conclusion that the latex plays two important roles in the whole process. Firstly, it acts as a capping agent even at the lowest concentration studied here. And secondly, the reduction of Zn(CH_3_COO)_2_ by NaOH is effectively catalyzed by the latex at higher concentrations. Since the latex effectively accomplishes the capping action, the particle size of the ZnONP remains limited to 25 nm in all the cases, as indicated by the SPR luminescence peak appearing at the same wave length of 281 nm. Osman and Mustafa [12] have reported the synthesis of the ZnONP, applying a hydrothermal technique using 0.05 or 0.1 M Zn(CH_3_COO)_2_:NaOH at 1:1 ratio. They have reported that, depending on the concentration of 0.05 or 0.1 M, the average NP size is found to be 75 or 54 nm in diameter, respectively [12]. On the contrary, in our present study, we used an extremely low concentration of the reducing agent (NaOH) so that the reduction reaction could be precisely controlled and the NPs could be effectively capped by the latex, limiting growth not beyond 25 nm.

### 4.2. ZnONPs Induce Oxidative Stress

Zinc is an essential micronutrient for plants, but, when in excess, Zn^2+^ causes oxidative stress in plants [38] even though the element is not redox-active. The ZnONPs are shown to be phytotoxic, as screened by the *Arabidopsis thaliana* [39], *Trticum aestivum* [7], and *Brassica nigra* [40] seedling assays. Similar to its antibacterial action, the phytotoxicity of ZnONPs is also attributed to the ROS generation, release of Zn^2+^, cellular membrane dysfunction, and NP internalization [41]. An oxidative stress signature, comprising multiple oxidative stress-related cellular, molecular, and metabolic events, is characteristic of the disturbance of redox homeostasis and signaling. 

In the present study, Zn(CH_3_COO)_2_ solutions at different concentrations were included as the positive control, where Zn^2+^ ions served as the source for Zn-toxicity to the root system. In the current study, the ZnONP-GS synthesized via the green route was thoroughly and repeatedly washed to ensure that it was free from any residual Zn^2+^ ions. This was confirmed by measuring the conductivity (a measure of Zn^2+^) of the supernatant of the experimental solutions of the ZnONP-GS at different concentrations (0–100 mg·L^−1^). In a control experiment, the conductivity was measured in pure Zn(CH_3_COO)_2_ solutions at different concentrations, wherein the conductivity appeared to increase with the increase in concentration of Zn(CH_3_COO)_2_. This confirms that the presence of Zn^2+^ ions in solution can be detected by conductivity measurements. Our conductivity measurements of the supernatant solutions over the ZnONP-GS showed no increase in conductivity over pure solvent (distilled water), indicating the absence of free Zn^2+^ ions that would contribute to the toxicity. The results suggested that Zn^2+^, and to a lesser extent the ZnONPs, enhanced the generation of ROS (O_2_^•−^, H_2_O_2_ and ^•^OH) (Figure 6), which is indicative of the membrane damage marked by the cell death and lipid peroxidation in the roots of germinating *L. sativus* seeds (Figure 7). The oxidative stress was further corroborated by the observed dose-dependent decrease of activity of CAT and the increase in the activities of SOD, GPX, and APX, induced by Zn^2+^ and the ZnONPs (Figure 8). The changed activities of these antioxidant enzymes serve as the markers of oxidative stress [42]. The Zn^2+^-induced decline in CAT activity with concomitant increases in the activities of SOD, GPX, and APX have been reported in studies on duckweed [43] and maize [44]. Among the antioxidant enzymes, the first enzyme is CAT, which catalyzes the dismutation of two molecules of H_2_O_2_ into water and oxygen, is very sensitive to O_2_^•−^, and is inactivated by the elevated levels of O_2_^•−^ [45]. The second enzyme, SOD, catalyzes the dismutation of O_2_*^•^*^−^ to O_2_ and H_2_O_2_. The third enzyme GPX, an effective quencher of ROS and peroxy redicals under stress conditions, showing a correlation with sub-lethal doses of metals, is considered a marker of metal toxicity [42]. The last enzyme investigated in the present study, APX, is regarded as one of the most widely distributed antioxidant enzymes in the plant cells and has greater affinity for H_2_O_2_ than CAT, thus making APX as an efficient scavenger of H_2_O_2_ under stress [42]. As compared to the ZnONPs, Zn^2+^ was more effective at altering the activities of the antioxidant enzymes in the present study. Thus, the results of the present study underscored the importance of the ZnONPs-induced ROS generation and oxidative stress as valid biomarkers of the ZnONP-mediated phytotoxicity assay. 

### 4.3. ROS Scavengers Alleviate ZnONP-Induced DNA Damage

From the standpoint of environmental health, Remedios et al. emphasize the need for more information on the genotoxicity of metal NPs, which is reported by the plant bioassays as well as the animal and human cell lines [46]. The ZnONPs have been reported to induce cytotoxicity, genotoxicity, and/or DNA damage in the root meristem cells of *Allium cepa* [47]; DNA damage in the epidermal cell line (A431) [48] and human neuroblastoma SHSY5Y cell line [49]; and somatic mutation and DNA damage in *Drosophila melanogaster* [50]. Also, Zn^2+^ has been shown to cause significant DNA damage in *Nicotiana tabacum* in the root but not leaf cells, attributed to efficient H_2_O_2_ scavenging by the enhanced antioxidative enzymes activity in leaf cells [51]. From the results of the majority of earlier reported studies, genotoxicity and DNA damage have been attributed to the oxidative stress induced by Zn^2+^ or ZnONPs. In the present study, it was clearly evident that Zn^2+^ and the ZnONP significantly induced DNA damage, wherein the former was more effective than the latter (Figure 9). The ROS scavengers, Tiron and DMTU, were used to establish the role of ROS i ZnONP-induced DNA damage and cell death. The concentrations of ROS scavengers were kept low to avoid their toxic actions. The ROS scavengers at such low concentrations were not expected to completely block the DNA damage or cell death but to alleviate toxicity of the ZnONPs. However, the priming of the roots with Tiron and DTMU (20 μM) significantly alleviated the cell death and DNA damage induced by the ZnONP alone (Figure 10), suggesting an underlying role for ROS in ZnONP-induced cell death and DNA.

## 5. Conclusions

From the results of the present study, it was concluded that the ZnONP-GS synthesized from Zn(CH_3_COO)_2_ by the alkaline precipitation method using the milky latex of *C. gigantea* induced oxidative stress in the root assay system of *L. sativus*, which was comparable to that induced by the commercially available ZnONP-S but to a lesser extent than that induced by Zn^2+^ alone. The findings underscore the role of ROS in ZnONP-induced DNA damage.
